# The effect of postoperative immobilization on short-segment fixation without bone grafting for unstable fractures of thoracolumbar spine

**DOI:** 10.4103/0019-5413.41870

**Published:** 2009

**Authors:** SH Lee, DS Pandher, KS Yoon, ST Lee, Kwang Jun Oh

**Affiliations:** Department of Orthopedics, Konkuk University Hospital, Konkuk University School of Medicine, 4-12 Hwayang-dong, Kwangjin-gu, Seoul 143-914, Korea; 1 Department of Orthopedics, Oxford Super-specialty Hospital, Jallandhar, India

**Keywords:** Delayed ambulation, dorsolumbar spine fracture, posterior fixation, short segment *vs* long segment

## Abstract

**Background:**

Controversy regarding the fixation level for the management of unstable thoracolumbar spine fractures exists. Often poor results are reported with short-segment fixation. The present study is undertaken to compare the effect of fixation level and variable duration of postoperative immobilization on the outcome of unstable thoracolumbar burst fractures treated by posterior stabilization without bone grafting.

**Patients and Methods:**

A randomized, prospective, and consecutive series was conducted at a tertiary level medical center. Thirty-six neurologically intact (Frankel type E) thoracolumbar burst fracture patients admitted at our institute between February 2003 and December 2005 were randomly divided into three groups. Group I (*n* = 15) and II (*n* = 11) patients were treated by short-segment fixation, while Group III (*n* = 10) patients were treated by long-segment fixation. In Group I ambulation was delayed to 10th-14th postoperative day, while group II and III patients were mobilized on third postoperative day. Anterior body height loss (ABHL) percentage and increase in kyphosis as measured by Cobb's angle were calculated preoperatively, postoperatively, and at follow-up. Denis Pain Scale and Work Scales were obtained during follow-up.

**Results:**

Mean follow-up was 13.7 months (range 3-27 months). At the final follow-up the mean ABHL was 4.73% in group I compared with 16.2% in group II and 6.20% in group III. The mean Cobb's angle loss was 1.8° in group I compared with 5.91° in group II and 2.3° in group III. The ABHL difference between groups I and II was significant (*P* = 0.0002), while between groups I and III was not significant (*P* = 0.49).

**Conclusion:**

The short-segment fixation with amenable delayed ambulation is a valid option for the management of thoracolumbar burst fractures, as radiological results are comparable to that of long-segment fixation with the advantage of preserving maximum number of motion segments.

## INTRODUCTION

The thoracolumbar segment of spine is an unstable zone between the fixed thoracic and mobile lumbar spine, and the goal of surgical management is the attainment of normal spinal anatomy, maintenance of reduction, decompression of neurological structures, and preservation of mobile segment for better long-term results. The optimal treatment of thoracolumbar unstable fractures is still a controversial issue.^1^,^2^ Many authors have demonstrated multilevel instrumentation techniques both in distraction and compression,^3^ while others have proposed shorter constructs to maintain motion segments, particularly in the lumber spine.^4^,^5^ The surgical treatment of the thoracolumbar spine has undergone profound changes in the past decade with an emphasis on the preservation of the intact segment (i.e., short-segment fixation).^6^ Good results have been reported in flexion distraction injuries;^7^ however, some studies have reported unacceptable results with short-segment fixation.^8^–^12^

Bone grafting is often added to any type of spine fixation, but it is considered that if the reduction is achieved soon after the injury, sufficient bone and soft tissue healing would occur to obviate the need for bone grafting as is the case with internal fixation for the limb fractures.^13^

The aim of this study is to evaluate the effect of delayed ambulation on short-segment fixation and to compare the results with long-segment fixation, without bone grafting in either group.

## PATIENTS AND METHODS

The study included 36 neurologically intact (Frankel Type E) patients of thoracolumbar spine injury admitted to our department between February 2003 and December 2005, who were randomly divided into three groups. Group I (*n* = 15) and II (*n* = 11) patients were treated by short-segment fixation, while group III (*n* = 10) patients were treated by long-segment fixation. The indications for surgery were unstable burst fracture, local kyphosis more than 20°, and anterior body collapse more than 40% of the vertebral height or more than 30% of canal area involvement. Demographic information, comorbid conditions, other injuries, and neurologic status were recorded. Plain radiographs and MRI were routine preoperative investigations. Additional CT scan was done whenever needed. Patients were randomly divided into three groups, using random number tables. In all the cases, pedicle screws were used for stabilization, and no bone grafting was done. Preoperative radiographic review included an assessment of the stability of fracture, according to classification given by McAfee *et al*. Local kyphosis was determined by measuring the Cobb angle^14^ between the superior end plate of the upper and the inferior end plate of the lower noninjured vertebrae.^15^ For the calculation of percentage of anterior body height loss (ABHL), anterior body height of the injured and the noninjured adjacent vertebrae above and below were measured, and the ABHL percentage was calculated using the formula adopted by Mumford *et al*.^16^ All the operations were performed by a senior author [SHL]. The operative details, follow-up findings, and complications if any were recorded. In group I, ambulation was delayed to 10th-14th postoperative day, while group II and III patients were mobilized on third or fourth postoperative day. At each follow-up, the patient was assessed for Denis Pain Score^17^ and neurological status. Radiographs were taken and evaluated for progress of union, local kyphosis, ABHL percentage, and instrumentation stability. Failure was defined as implant failure or an increase of 10° or more in local kyphosis in the latest follow-up radiographs compared with the measurement on the initial postoperative radiographs.^11^ Radiographic reviewers were blinded to the functional outcome of the patient and the time of follow-up. The Denis Work Status Assessment Scale^17^ was used to record the functional outcome at follow-up. This provided the most recent evaluation of a patient's back-related postoperative disability.

### Surgical technique

All patients were operated under general anesthesia in prone position on commercially made prone bar. A midline posterior approach was used. The extent of the injury was defined. Posterior decompression was performed whenever indicated to ensure that the disrupted soft tissues or bone fragment did not compress neural elements during final reduction.^18^ Decompression also included undercutting the disrupted lamina and evacuation of any epidural hematoma from the enlarged epidural space. The facets and other bony structures were used to judge an anatomic reduction. Depending on the preoperative random grouping, long-segment or short-segment fixation was performed, and the fracture was stabilized with bilateral pedicle screws under fluoroscopy control. For short-segment fixation, only the injured motion segment was stabilized. For long-segment stabilization, two segments above and below the injured vertebra were engaged with pedicle screws. Distraction was used to reduce the fragments of fractured vertebra, by the principle of ligamentotaxis. No bone grafting was done in any group. Postoperatively all patients with long-segment fixation and group II of short-segment fixation were mobilized on third or fourth day with thoracolumbar spinal brace. In group I of short-segment fixation, patients ambulated on 10^th^-14^th^ postoperative day. The spinal brace was worn for three months postoperatively in all groups.

### Statistical analysis

ANOVA and Student's *t*-tests were performed for statistical analysis to determine comparability of different groups and to compare the final results. Significance was set at *P* < 0.05.

## RESULTS

There were 23 men and 13 women in our study, with the mean age of 45.7 years (±12.9, range 21-83 years) [Table 1]. The groups were analyzed for between-group comparability regarding demographic variables of patients and were found comparable according to ANOVA (*P* = 0.48). Mode of injury was fall from height (n=24), slip (n=6), direct trauma (n=4), car accident and pedestrian accident (n=1). The level of the fracture was L_1_ in 15 patients, L_3_ in seven patients, L_2_ in six patients, T_12_ in five patients, L_4_ in two patients, and T_8_ in one patient. According to McAfee classification, all fractures were of unstable burst type. Two patients had associated chance fracture of adjacent vertebra, which was managed adequately. These fractures were not included in the analysis of data. The average time from injury to operation was 2.78 days (±2.13 days, range 0-7 days). The average time of surgery was 90 minutes (±10 min, range 75-110 min). Mean follow-up duration was 13.74 months (±5.58, range 3-27 months).

**Table 1:** Showing McAfee classification, level of fracture, anterior body height loss (%), Cobb's angle (°), Denis Pain and Work Scale, and follow-up duration in three groups

Number	Sex/age	McAfee/level	Anterior body height loss (%)			Cobb's angle (°)			Denis scale		Follow-up (months)
						
			Pre-operative	Post-operative	Follow-up	Pre-operative	Post-operative	Follow-up	Pain	Work	
Group I											
1	M/44	unstable burst fx/L2	40	0	7	6K	4K	5K	P2	W2	6
2	M/50	unstable burst fx/L1	24	10	14	7K	5K	5K	P3	W3	7
3	M/54	unstable burst fx/L4	27	8	8	27L	31L	28L	P2	W2	8
4	M/42	unstable burst fx/L2	42	0	8	20K	3K	5K	P2	W2	9
5	F/52	unstable burst fx/L2	32	10	10	18L	16L	16L	P2	W2	10
6	F/49	unstable burst fx/L3	30	0	0	20L	16L	16L	P2	W2	10
7	F/22	unstable burst fx/L1	55	20	23	20K	12K	16K	P1	W2	10.5
8	M/52	unstable burst fx/L3	28	6	8	22L	10L	16L	P2	W2	11
9	M/51	unstable burst fx/T12	40	10	20	30K	12K	14K	P2	W2	11
10	F/53	unstable burst fx/L3	30	6	6	20L	15L	15L	P2	W2	12
11	M/50	unstable burst fx/L1	24	10	18	7K	5K	6K	P2	W2	14
12	M/52	unstable burst fx/L1	24	5	7	10K	8K	10K	P2	W3	15
13	F/46	unstable burst fx/T8	52	20	24	23K	22K	23K	P3	W3	16.5
14	M/50	unstable burst fx/L3	32	6	18	20L	17L	20L	P2	W3	19
15	F/58	unstable burst fx/L1	24	7	12	10K	8K	12K	P2	W3	20.5
Group II										
1	M/70	unstable burst fx/L1	10	10	45	20K	2K	18K	P3	W3	6
2	F/40	unstable burst fx/L3	40	4	28	20L	10L	18L	P3	W3	8
3	F/29	unstable burst fx/L1	36	8	22	20K	6K	16K	P2	W2	10.5
4	M/42	unstable burst fx/L3	30	6	20	20L	16L	20L	P2	W2	11
5	M/24	unstable burst fx/T12	60	29	32	30K	21K	22K	P2	W2	12
6	M/40	unstable burst fx/L1	40	12	20	20K	6K	10K	P2	W2	12.5
7	M/21	unstable burst fx/T12	40	30	35	20K	17K	16K	P2	W2	13
8	F/83	unstable burst fx/L2	42	10	30	6K	2K	5K	P3	W3	14
9	F/38	unstable burst fx/L4	28	8	24	27L	31L	28L	P3	W3	15
10	M/46	unstable burst fx/T12	60	20	42	30K	18K	26K	P3	W2	15
11	M/29	unstable burst fx/L1	38	20	37	20K	12K	19K	P3	W3	18
Group III										
1	M/49	unstable burst fx/L1	36	29	29	12K	7K	8K	P2	W3	3
2	M/26	unstable burst fx/L1	40	27	27	22K	0	10K	P2	W2	12
3	F/38	unstable burst fx/L2	40	4	10	6K	4K	5K	P2	W2	15
4	M/49	unstable burst fx/L1	45	20	30	16K	12K	15K	P2	W3	16
5	M/58	unstable burst fx/T12	37	18	18	29K	18K	19K	P2	W3	17
6	M/42	unstable burst fx/L3	44	6	8	24L	16L	16L	P2	W2	18
7	M/36	unstable burst fx/L1	42	12	22	20K	7K	9K	P2	W2	22
8	M/43	unstable burst fx/L2	41	7	10	7K	3K	4K	P2	W2	24
9	F/57	unstable burst fx/L1	31	4	25	10K	5K	8K	P3	W3	26
10	F/59	unstable burst fx/L1	31	10	20	11K	6K	7K	P2	W2	27

The preoperative ABHL percentage and Cobb angle was similar in three groups according to anova (*P* = 0.37 and 0.22, respectively). The difference between preoperative and postoperative values of anterior body height loss and Cobb's angle for three groups was also not significant according to anova (*P* = 0.92 and 0.26, respectively); that is, all the groups had similar degree of correction after surgery for these indices.

At the final follow-up, the mean ABHL percentage was 4.73% (±3.88%, range 0-12%) in group I compared with 16.2% (±9.16%, range 3-35%) in group II and 6.20% (±6.71%, range 0-21%) in group III. The ABHL difference between group I and II was significant (*P* = 0.0002), while the difference between groups I and III was not significant statistically (*P* = 0.49). The mean wedge angle loss was 1.8° (±1.7°, range 0-4°) in group I compared with 5.91°(±4.48°, range 1°-16°) in group II and 2.3°(±2.87°, range 0-10°) in group III. The change in Cobb's angle between groups I and II was significant (*P* = 0.0033), while the difference between groups I and III was not significant statistically (*P* = 0.59).

Mean Dennis Pain Scores for groups I, II, and III were 2.07, 2.55, and 2.10, respectively. The results of group I were significantly better than that of group II (*P* = 0.02; Student's *t*-test), whereas the results of group III were similar to group I (*P* = 0.84; Student's *t*-test). Mean Dennis Work Scores for groups I, II, and III were 2.07, 2.55, and 2.10, respectively. The results of group I were not significantly better than that of group II (*P* = 0.55; Student's *t*-test), and the results of group III were similar to that of group I (*P* = 0.75; Student's *t*-test).

**Figure 1:**
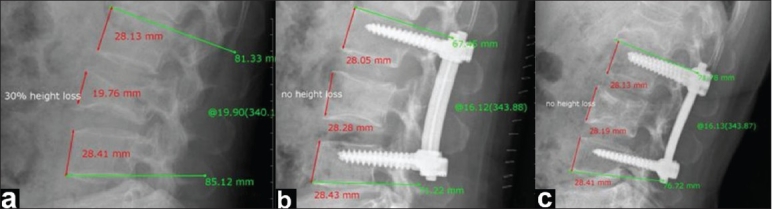
Group I case: ABHL percentage and kyphosis angles as measured by PVR system for X-ray viewing: (a) Preoperative X-ray showing 30% ABHL and 20° lordotic angle, (b) Postoperative X-ray showing no ABHL and 16° lordotic angle, (c) Follow-up X-ray showing no ABHL and 16° lordotic angle

**Figure 2:**
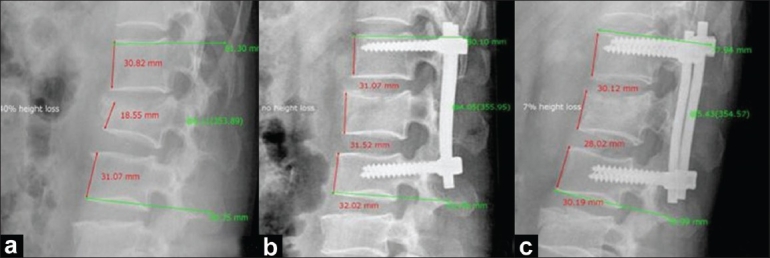
Group I case: ABHL percentage and kyphosis angles as measured by PVR system for X-ray viewing: (a) Preoperative X-ray showing 40% ABHL and 6° kyphotic angle, (b) Postoperative X-ray showing no ABHL and 4° kyphotic angle, (c) Follow-up X-ray showing 7% ABHL and 5° kyphotic angle

**Figure 3:**
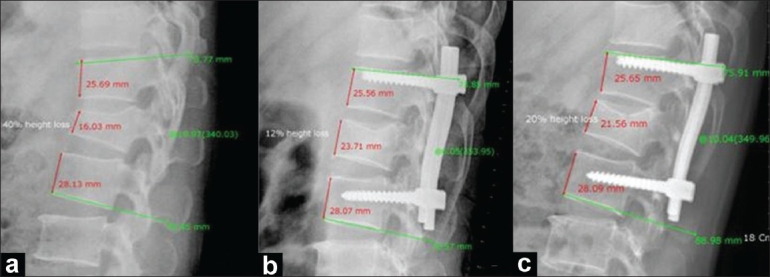
Group II case: ABHL percentage and kyphosis angles as measured by PVR system for X-ray viewing: (a) Preoperative X-ray showing 40% ABHL and 20° kyphotic angle, (b) Postoperative X-ray showing 12% ABHL and 6° kyphotic angle, (c) Follow-up X-ray showing 20% ABHL and 10° kyphotic angle

Thus the results clearly indicate that delayed ambulation group of short-segment fixation group [Figures 1 and 2] has outcome similar to long-segment fixation, and it is significantly better than early ambulation group of short-segment fixation [figure 3]. Descriptive statistics summarizing the Cobb's angle and percentage anterior body collapse are given in [Table 2], and Denis Pain Scale and Work Scale^17^ outcomes are shown in [Table 1].

**Table 2:** Showing postoperative correction and final follow-up loss of correction of anterior vertebral body height (%) and Cobb's angle (°)

	Pre- and postoperative difference	Postoperative follow-up reduction loss
Anterior vertebral body height (%) (mean ± SD, range)		
Group I	26.3+/−8.94	4.73+/−3.88
Group II	24.8+/−10.5	16.2+/−9.16
Group III	25.0+/−10.1	6.20+/−6.71
*P* value (I and III)	0.74	0.49
*P* value (I and II)	0.71	0.0002
Cobb's angle (°) (mean ± SD, range)		
Group I	5.53+/−5.63	1.8+/−1.7
Group II	9.09+/−5.03	5.91+/−4.48
Group III	7.90+/−6.01	2.3+/−2.87
*P* value (I and III)	0.33	0.59
*P* value (I and II)	0.11	0.0033

There were no neurological complications related to surgery, or more specifically to pedicle screw placement. There were no wound infections. In two cases of long-segment fixation, implant removal was required, as there was a risk of skin breakdown due to the irritation by the rods. We had one case of screw breakage in group II; however, since the breakage occurred after bony union was achieved; it did not affect the clinical outcome. The screw breakage was a chance finding on routine follow-up X-ray. None of our patients required revision surgery for back pain or deformity. Any medical complications arising from delayed ambulation were not reported in our study.

## DISCUSSION

The debate between the operative and nonoperative treatment of thoracolumbar spine in view of cost effectiveness exists. Siebenga *et al*.^19^ demonstrated that, in the treatment of traumatic thoracolumbar spine fractures, the indirect costs exceed the direct costs by far and make up 95.4% of the total costs for treatment in nonsurgically treated patients compared with 71.6% of the total costs in the operative group, and in view of cost-effectiveness, the operative therapy of traumatic thoracolumbar spine fractures is to be preferred. The optimal surgical treatment of thoracolumbar burst fractures still remains controversial. Combined anterior-posterior approach allows for three column reconstruction and stabilization,^20^ but the time needed for surgery and morbidity rates associated are much higher than a single approach. Anterior approach has proven effective in allowing for extensive decompression and successful fusion with minimal loss of sagittal alignment,^21^–^23^ but it also requires longer surgery time and has higher rates of surgery-related morbidity associated with an anterior spinal exposure when compared with the posterior approach.^23^ Posterior fixation has become a popular method in the treatment of thoracolumbar burst fractures after the introduction of transpedicular screws.^24^–^27^ Extension of the posterior pedicle screw-rod construct to include two or more segments above and below the fractured level can reduce instrument failure rates, but it also sacrifices additional motion segments and ultimately reduces the range of motion.^28^ Preservation of motion segments is desired if possible,^7^ as a short-segment fixation results in less spinal stiffness^29^ and patients perform comparatively better.^30^ In lumbar spine fractures, short fixation is the best option because loss of lumbar lordosis associated with flat back syndrome can be avoided.^31^,^32^ Advantage of short-segment pedicle screw fixation is that it potentially allows for spinal stabilization while preserving as many motion segments as possible. Immobilization of long segment increases the load and motion not only at the immediate adjacent segment, but also at the distal segments,^33^ which has detrimental effect in the long term. Forces applied to the spine in short-segment fixation are not strong, and fatigue failure is uncommon.^34^,^35^ Moreover, it is not necessary to remove the construct, although this is often required with long rods. Because of the above mentioned advantages, short-segment fixation is gaining popularity as a preferred mode of fixation for thoracolumbar spine fractures, and various additional procedures along with short-segment fixation are being reported in recent literature.^36^–^38^

Bone grafting is often performed as an adjunct to spinal fixation, but there are several advantages in not performing fusion, such as reduced surgery time and blood loss, often in critically injured patients.^13^ Bone grafting is not without complications; Frymoyer *et al*. in a long-term study reported that 37% of patients identified donor site pain as a problem 10 or more years after operation.^39^ advantage is that the facet joints adjacent to the fracture are less disturbed, because surgical soft tissue stripping required to prepare the bone graft bed is no longer needed.^13^ In a recent prospective randomized study, Alanay *et al*.^40^ found no difference in the failure rates of short-segment pedicle screw constructs supplemented with transpedicular intracorporeal bone graft compared with the constructs without graft support. Moreover, transpedicular graft material can cause spinal canal compression if not placed properly.^41^ Knop *et al*. also could not show any advantage of intra- and intercorporeal grafting in their series.^41^ Knop *et al.* also could not show any advantage of intra- and intercorporeal grafting in their series.^42^

In our study, we included patients with no neurological deficit only, as we believe that the homogeneity of the neurologic status among the study groups may also be important. The loads acting on the reconstructed injured vertebrae may be different for a bedridden patient with paraplegia compared to a neurologically intact mobile patient. Our results demonstrated that after a substantial initial correction, there was a gradual partial loss of anterior body height and Cobb's angle in all the groups including delayed ambulation short-segment fixation, which seems largely because of the loss of disc height. This loss of initial correction has been reported by other authors who have routinely fused the spine, with some of them reporting a more marked correction loss than that in our series.^43^–^46^ Our mean follow-up was 13.7 months, which is sufficient to rule out any further collapse, and the critical period according to Parker *et al*.^30^ seems to be six months.

The radiographic evaluation of the fractured vertebral segment at final follow-up showed that the increase in kyphosis from postoperative value was significant in group II, but the angle change in groups I and III was less pronounced. The difference between results of the latter two groups was also not significant. This clearly shows that though early ambulation in short-segment fixation has less satisfactory results as proved by many other studies,^9^,^11^,^40^ but results similar to long-segment fixation can be achieved by delayed ambulation in short-segment fixation.

Tezeren and Kuru demonstrated similar clinical outcome of short-segment fixation and long-segment fixation, though their radiographic findings demonstrated that short-segment pedicle instrumentation had a high failure rate, i.e., >10° loss of correction, as compared with long-segment fixation.^47^ But, because the clinical outcome data did not show any difference between the short-segment and long-segment instrumentation, should the failure as defined by the author considered as a true failure for the patient? In our study, we also set 10° loss of correction as a criteria for failure, but it did not correlate well with clinical findings in two cases, which had more than or equal to 10° loss of correction. In this study,^47^ the results are poor in short-segment fixation, as the postoperative ambulation was started on third or fourth day in both groups. The fact that our study highlights is that if ambulation is delayed in short-segment fixation, results can be rewarding.

Moon *et al.* demonstrated that short-segment fixation without posterolateral fusion is an effective procedure for compression and burst fractures, which contributes to the fractured vertebral body consolidation without recollapse and maintains the motion segment function.^48^ One limitation of their study was lack of comparison between early and delayed postoperative patients' mobilization and comparison with long-segment fixation, which is being addressed in our study.

The main limitation of our study is that it is a small series, but it is the only study of its kind that is randomized, prospective, and consecutive, highlighting the benefits of delayed ambulation in short-segment fixation. The final results of our study are statistically significant and favor amenable delayed ambulation as a preferred protocol for postoperative management of short-segment fixation. It gives results similar to long-segment fixation in terms of radiological indices while keeping all the benefits of short-segment fixation like less invasive surgical approach with less operating time and minimal soft tissue stripping around the injured vertebra thus maintaining the vascularity, which is most important factor for healing in any type of injury.

## CONCLUSION

The patient selection and postoperative compliance for delayed ambulation is the key stone to the success of short-segment fixation of thoracolumbar spine fractures treated by pedicle screw instrumentation without bone grafting, which definitely has advantage of more mobile segment preservation, with better functional results. The radiological results of short-segment fixation with delayed mobilization are similar to that of long-segment fixation.
